# Fighting Off Wound Pathogens in Horses with Honeybee Lactic Acid Bacteria

**DOI:** 10.1007/s00284-016-1080-2

**Published:** 2016-06-21

**Authors:** Tobias C. Olofsson, Éile Butler, Christina Lindholm, Bo Nilson, Per Michanek, Alejandra Vásquez

**Affiliations:** 1 Laboratory Medicine, Lunds Universitet, Lund, Sweden; 2 Division of Nursing Science, Sophiahemmet Hogskola, Stockholm, Sweden; 3 Laboratory Medicine, Clinical Microbiology, Region Skåne, Lund, Sweden; 4 Department of Laboratory Medicine Lund, Medical Microbiology, Lund University, Sölvegatan 23, 22362 Lund, Sweden; 5 Animal Farm Veterinary Consultants, Degebergavägen, 27568 Vollsjö, Sweden

## Abstract

In the global perspective of antibiotic resistance, it is urgent to find potent topical antibiotics for the use in human and animal infection. Healing of equine wounds, particularly in the limbs, is difficult due to hydrostatic factors and exposure to environmental contaminants, which can lead to heavy bio-burden/biofilm formation and sometimes to infection. Therefore, antibiotics are often prescribed. Recent studies have shown that honeybee-specific lactic acid bacteria (LAB), involved in honey production, and inhibit human wound pathogens. The aim of this pilot study was to investigate the effects on the healing of hard-to-heal equine wounds after treatment with these LAB symbionts viable in a heather honey formulation. For this, we included ten horses with wound duration of >1 year, investigated the wound microbiota, and treated wounds with the novel honeybee LAB formulation. We identified the microbiota using MALDI-TOF mass spectrometry and DNA sequencing. In addition, the antimicrobial properties of the honeybee LAB formulation were tested against all wound isolates in vitro. Our results indicate a diverse wound microbiota including fifty-three bacterial species that showed 90 % colonization by at least one species of *Staphylococcus.* Treatment with the formulation promoted wound healing in all cases already after the first application and the wounds were either completely healed (*n* = 3) in less than 20 days or healing was in progress. Furthermore, the honeybee LAB formulation inhibited all pathogens when tested in vitro. Consequently, this new treatment option presents as a powerful candidate for the topical treatment of hard-to-heal wounds in horses.

## Introduction

We are facing a global increase in bacterial resistance to conventional antibiotics that makes researchers around the world investigate alternative tools to reduce this need in both human and animal infection [60]. There are many reasons for this antibiotic resistance crisis both from the bacterial resistance mechanism and societal perspective, but a significant issue is due to the therapeutic and prophylactic over-use of antibiotics in pharmaceutical, dairy, and food industry and the huge volumes of waste that is generated from these industries [22, 37]. It is estimated in the US alone 23 × 10^6^ kg of antibiotics are used annually [31]. In the European Union, there is much restrictions on the use of antibiotics in agriculture and food industry yet in many cases, it varies vastly between countries [36]. In Sweden, it is mandate that antimicrobial growth promoters are completely restricted in food and animal production without veterinary prescription and only to cure or prevent disease [61]. However, due to the over-use in other areas of veterinary care such as over-prescription because of patient/owner demands or bacterial misdiagnosis [37], antibiotic resistance in the veterinary community is still occurring at an alarming rate in some cases [4, 48]. Chronic wound management is facing the same issues when it comes to finding potent topical antiseptics/antimicrobials that are sustainable, broad spectrum, cost effective, and environmentally friendly [32]. Due to the expenses associated with maintaining and keeping horses healthy and pleased, many owners are desperate to find suitable treatments for persistent wounds.

The physical nature of the horse and their natural outdoor habitat puts them at risk for many traumatic injuries, commonly skin and soft tissue wounds located on the limbs. Objects from their surrounding environment often cause these wounds, such as fences or gates. Contaminants from soil can often leads to colonization, infection and finally to disrupted healing [58]. In some cases, wound infection can lead to Pastern Dermatitis (mud fever) [16]. Wounds on the limbs of horses have many similarities with wound healing in humans. The hydrostatic forces of the limbs cause risk of micro- and even macro-edema similar to human leg ulcers, which compromises wound healing with the progression following the same phases in hard-to-heal human wounds such as inflammation, granulation, epithelialization, and contraction [17]. These phases are influenced by numerous factors, which can cause delayed healing of which the bio-burden is an important one [51]. Equine wounds tend to have a diverse environment of bacteria, similar to that of human chronic wounds, with many different species implicated in causing the infection, and possibly biofilm formation [59]. The treatment of equine wounds is becoming progressively difficult due to increase of antibiotic-resistant bacterial strains (e.g., Methicillin-Resistant *Staphylococcus aureus (*MRSA). This is explained by the mentioned over-use of antibiotics as well as a lack of appropriate topical wounds treatments [10, 50, 57].

For thousands of years, honey has been used as a folk medicine in treating infections [19, 29, 33, 46], and now, it is being investigated further in treating wound infections in humans [26] and animals [13]. The antimicrobial activity of honey is largely attributed to its hygroscopic nature, high osmolality, low pH, hydrogen peroxide content [26, 30], and in the case of Manuka honey (one of the most widely used in medicine), the antimicrobial substance Methylglyoxal (MGO) [1]. MGO is believed to originate in the nectar of the flowers of the Manuka tree (*Leptospermum scoparium*); however, this substance can also be produced by microorganisms [1, 8] including some lactic acid bacteria (LAB) species [20, 34]. Manuka honey was investigated in the treatment of Equine Pastern Dermatitis with some success [21], though other studies have shown that there may be other honey types that possess equal or similar healing properties in animal and human wounds, such as Heather honey [13] suggesting other mechanisms of action [30].

Previously Olofsson and Vásquez discovered a symbiotic group of LAB composed of nine *Lactobacillus* species, two *Bifidobacterium* species and two *Bifidobacterium* phylotypes currently undergoing description as novel species, found in the honey crop of the western honeybee *Apis mellifera* [39, 55]. Notably, although often referred to as LAB, *Bifidobacteria* are not typical representatives of LAB as their main product of fermentation is acetic acid, not lactic acid. These LAB symbionts, of which the majority were recently described as novel species [40], are involved in the production of honey and are viable in all types of freshly harvested honey in extraordinary concentrations (10^8^ LAB per gram of fresh honey) [54, 56]. Further investigations have been performed to reveal if these bacterial symbionts are the key reasons to honey’s antimicrobial and therapeutic properties independently of its geographic or nectar origin.

Today, it is known that the 13 LAB symbionts produce numerous extra-cellular proteins with a putative antimicrobial action during honey production [11, 49] that end up in mature honey showing for the first time an equal and standardized honey production by which honeybees produce their food [41]. Besides from the production of several putative antimicrobial proteins, these symbionts was shown to produce other substances including acetic and formic acid, 2-heptanone, 3-hydroxy fatty acids, and hydrogen peroxide that have antimicrobial and healing properties [41] important for any future wound application.

Historical application of honey as a wound healing folk medicine and recent research findings encouraged us to perform a trial on hard-to-heal wounds in horses with a standardized and previously used formulation. The antimicrobial and pro-healing substances produced by the LAB symbionts was reported often not being present in mature honey including medical grade types due to the non-viability of the LAB and the sensitive nature of the bioactive substances in honeys high osmotic environment [36]. The novel formulation therefore mimics fresh honey, with a controlled standardized amount of the viable LAB in a sterile honey matrix. It was recently tested in vitro for its antimicrobial activity against human pathogens isolated from 22 patients suffering from various chronic wound types, and the results showed that the honeybee LAB formulation was active against all isolates tested [37].

Since heavy bio-burden in wounds and chronic ulcers promotes a prolonged inflammatory process and sometimes counteracts healing [28, 53], we hypothesized that the documented synergistic antimicrobial and healing properties of the honeybee LAB symbionts observed in our previous laboratory studies would be an ideal tool to test in hard-to-heal wounds such as those seen in horses as a wound model.

Thus, there are three main aims of the present study. First, to identify the microbiota of hard-to-heal equine wounds in order to study the honeybee LAB formulation’s mechanisms of antimicrobial action. Second, to investigate if the honeybee LAB formulation could initiate wound healing in hard-to-heal equine wounds and to detect potential adverse effects. And finally, to investigate if this formulation can be a stepping-stone when finding new alternative tools in wound management for animals and/or humans.

## Method

### Ethics

Ethical approval (M 18–13, 6th March 2013) was obtained, regarding the use of the honeybee LAB formulation in horses by the Ethical Committee on Animal Experiments in Lund/Malmö, Sweden.

### Treatment Formulation

The honeybee LAB formulation used in this study was prepared as previously described [12] with some modifications. The mixture consisted of the 13 viable species of LAB: *Lactobacillus kunkeei* Fhon2, *Lactobacillus apinorum* Fhon13, *Lactobacillus mellifer* Bin4, *Lactobacillus mellis* Hon2, *Lactobacillus kimbladii* Hma2, *Lactobacillus melliventris* Hma8, *Lactobacillus helsingborgensis* Bma5, *Lactobacillus kullabergensis* Biut2, *Lactobacillus apis* Hma11, *Bifidobacterium coryneforme* Bma6, *Bifidobacterium* sp. Bin7, *Bifidobacterium asteroides* Bin2 and *Bifidobacterium* sp. Hma3 [9, 27, 40, 43] (total cell count of all 13 LAB; 10^9^ cfu/g honey), and their bioactive produced substances in a matrix of Swedish sterilized heather (*Calluna vulgaris*) honey. Sterilization of the honey was performed at 102 °C for 30 min resulting in disinfection killing of most microbial life except certain bacterial spores. To obtain a spray form, the same formulation was mixed with sterile (autoclaved) water (1 g/2 ml) and incubated at room temperature 1 day before treatment to promote the growth of LAB and their production of bioactive substances.

### Experimental Design and Sample Collection

Ten horses with hard-to-heal wounds (wound duration >1 year) diagnosed and pre-study treated by a veterinarian were included in this pilot study. Each horse owner had to fill in a protocol prior to the start of the study including horses’ age, breed, previous treatments, and wound duration (Table 1). All wounds had signs of clinical infection at study start. The horses’ age ranged between 6 and 23 years, and all wounds were previously treated with different topical agents without success (Table 1). Study period was 20 days or until healing if this occurred before 20 days.

**Table 1:** Participant information and treatment results about each horses included in this study, containing horse breed, age, wound duration, past treatments, underlying infection or disease, completed treatment, percentage/number of completely healed wounds per horse, time until wounds completely healed, and comments

Horse no.	Breed	Age	Wound size (^2^cm)	Wound duration (year, month)	Previous treatment	Underlying infection	Finished trial	Healed wounds (%, no.)	Time until healed (days)	Comments
H1:	Swedish warm-blood	>4	9 3	1 year	Chlorhexidine	Diagnosed with pastern dermatitis	Yes	100 (2/2)	20	Wound healed completely
H2:	Swedish warm-blood	23	NA	1 year	Chlorhexidine penicillin	Past case of lymphangitis	Yes	80 (4/6)	20	Four out of six wounds healed at day 20
H3:	North Swedish draft horse	6	10	2 years	Honey phoxim	No known disease or allergies	Yes	100 (1/1)	16, 16	Wound healed completely
H4:	Swedish warm-blood	7	1 0.5 5 5	2 years	Honey sulfadiazine fucidin	No known disease or allergies	Yes	75 (3/4)	10, 10, 10	Three wounds healed completely. 4th wound almost closed at day 20
H5:	Swedish warm-blood	8	All 9	1 year	Honey chlorhexidine fucidin	No known disease or allergies	Yes	50 (2/4)	20, 20	Two wounds healed completely. 3rd and 4th wound reduced in size with hair regrowth
H6:	Swedish warm-blood	6	–	1.5 years	Chlorhexidine metacam	No known allergies	No	–	–	Interrupted because treatment protocol was not followed
H7:	Danish warm-blood	17	6 6	1 year 7 months	Penicillin sulfadiazine zinc oxide cream	Shivering, no known allergies	Yes	100 (2/2)	10	Wound healed completely
H8:	Tinker mare	12	0.25 0.25 4 9	>3 year	Chlorhexidine antibiotic	No other allergies or diseases	No	0 (0/4)	–	Interrupted on the request of the owner due to painful wounds
H9:	Thorough bred	8	–	3 years	Lactacyl honey chlorhexidine	No other allergies or diseases	No	–	–	Interrupted on the request of the owner due to painful wounds
H10:	Swedish warm-blood	12	100	4.5 years	Chlorhexidine hydrogen peroxide	No other allergies or diseases	Yes	0 (0/1)	Still healing at day 20	Wounds had reduced size at day 20

The horse owners described and observed the treated areas during the time of treatment. The wounds were first visually judged, measured for size and photographed, cleansed with saline solution, and then microbiological samples were taken with a transport swab containing charcoal (Sarstedt, Sweden) of the infected area for microbial analyses both before and after treatment. The honeybee LAB formulation was applied in original gel form and as diluted with sterile water to a spray. It was applied to the entire wound and covered with bandage. The gel was applied directly to the moist wound. The spray was used in the exudative parts of the wounds [25]. The wounds were treated every 2 days and protocol data were recorded at the same time. Data included clinical scores for inflammation and healing, and signs of infection (smell, pain, swelling, exudation, hyper-granulation and necrotic tissue).

### Bacterial Culture

Wound samples were received as swabs (described above) 1 day after sample were taken. A dilution series was made using sterile PBS (pH 7.2), and samples were inoculated onto tryptone soy broth agar (Oxoid, Basingstoke, Hampshire, England) plates supplemented with, respectively, horse blood and haematein for aerobic incubation and onto equal plates and fastidious anaerobe agar (FAA, Oxoid) plates, supplemented with horse blood, for the anaerobic incubation at 37 ^°^C for up to 48 h. All colonies were counted (total counts), and morphologically different colonies were then picked for further identification.

### Matrix-Assisted Laser Desorption Ionization–Time of Flight (MALDI-TOF) Mass Spectrometry (MS)

MALDI-TOF MS was performed for the identification of isolated microorganisms from horse wounds as previously described [12, 44] with few modifications. Bacterial isolates were cultured as described above and the direct transfer formic acid method was used for all samples [44]. The experiments were performed in linear-positive mode on Ultraflextreme MALDI-TOF/TOF MS instrument (Bruker, Sweden) in a mass range of 2–20 kDa. Mass spectra were analyzed using the FlexControl and MALDI Biotyper 3.1 software with the BDAL-5627 reference database (Bruker Daltronics, Sweden). Samples that were not identified by MALDI-TOF MS were prepared for 16S rRNA gene polymerase chain reaction (PCR) amplification and sequencing.

### 16S rRNA Genotypic Characterization

Characterization of unidentified organisms using 16S rRNA gene sequencing was carried out according to previous work [39] with some modifications and described briefly here. Colonies of each unidentified organism were re-cultured for 24–48 h depending on their growth conditions. DNA was extracted by bead extraction (Sigma-Aldrich, USA) before PCR amplification of 16S rRNA genes. One colony from the purified isolates was placed in 2.0 ml Eppendorf tubes together with 0.25 ml sterile water and 10–15 glass beads (2.0 mm). Cells were disintegrated by shaking for 45 min in an Eppendorf mixer 5432 (Eppendorf, Hamburg, Germany). After centrifugation (20,200×*g* for 5 min), 1 µl of the supernatant was used in the following PCR reaction. Amplification of isolates was performed using universal primers ENV1 and ENV2 (TAG, Copenhagen, Denmark) designed to anneal to conserved regions of bacterial 16S rRNA genes. The forward primer ENV1 (5′-AGA GTT TGA TII TGG CTC AG-3′) corresponded to positions 8–27 of *Escherichia coli* 16S rRNA, and the reverse primer ENV2 (5′-CGG ITA CCT TGT TAC GAC TT-3′) corresponded to positions 1511–1492. The PCR reaction contained 5 µl ten PCR buffer (100 mmol/l Tris–HCl, 15 mmol/l MgCl2, 500 mmol/l KCl, pH 8.3), 200 µmol/l of each deoxyribonucleotide triphosphate, 2.5 U of Taq DNA polymerase (Roche Diagnostics, Mannheim, Germany), 10 pmol of each primer, and 1–10 µl template in a total volume of 50 µl. Unpurified PCR products were sent for Value Read sequencing at Eurofins MWG operon (Ebersberg, Germany), and sequences were then searched against GenBank (National Centre for Biotechnology Information (NCBI), Rockville Pike, MD) using the advanced BLAST similarity search option (available at http://www.ncbi.nlm.nih.gov).

### Dual Culture Overlay Assay

Antimicrobial activity was measured by using dual culture overlay assay as previously described [12, 35] with few modifications. Honeybee LAB formulation in spray form (10 µl, 10^8−10^ cfu/disk) was added into a filter disk and placed on de Man, Rogosa & Sharpe (MRS) (supplemented with 0.1 % l-cysteine and 2 % fructose) agar plates followed by overnight incubation at 35 °C. Positive quality controls strains (Culture collection isolates from American Type Culture Collection (ATCC) of common wound pathogens; *S. aureus* ATCC29213, *E. coli* ATCC 25922, *Staphylococcus epidermidis* ATCC14990 and *Proteus vulgaris* ATCC13315) and the identified wound pathogen cultures were mixed with a 10 ml soft agar (0.8 %), containing their respective growth medium, holding a temperature of 42 °C. Prior to mixing, pathogenic cultures were adjusted to 10^8^ cells per ml (OD of 0.5–0.6 at 540 nm). Each mixture of soft agar was poured as an over layer on top of supplemented MRS plates with the overnight cultivated LAB formulation. The plates were incubated at 37 °C for 24–48 h. Zone diameters were measured from center of disk to zone edge and doubled for diameter.

## Results

### Wound Healing

**Fig. 1:**
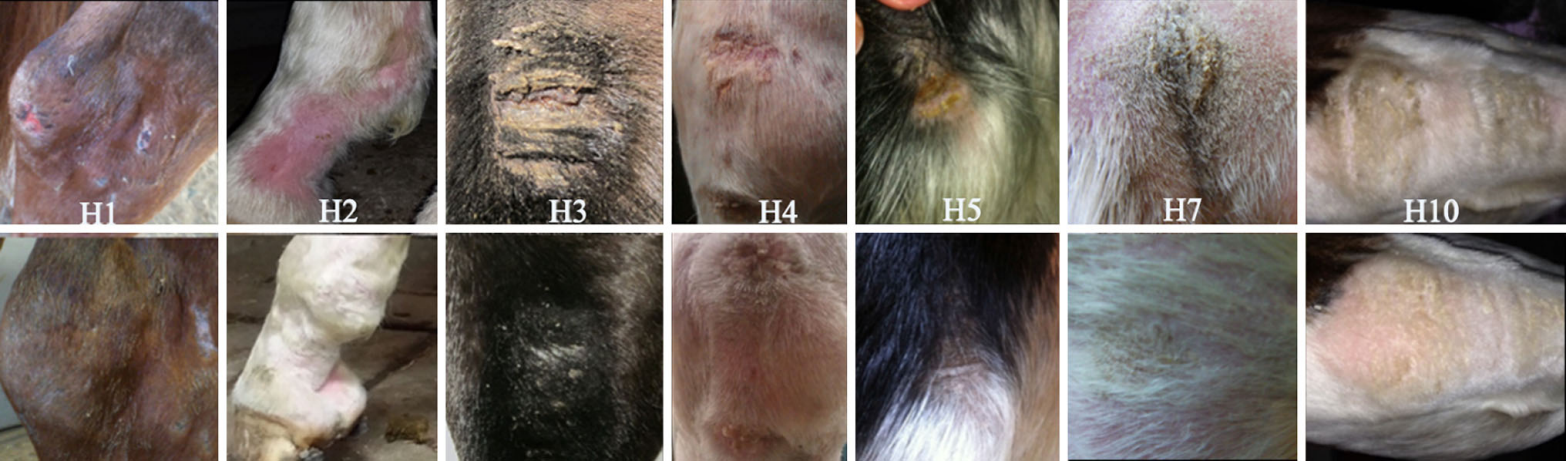
Pictures before treatment (*top*) and after (*bottom*) treatment of the wounds for the seven horses that completed the study with the honeybee LAB formulation. The horse owners took photos as outlined in protocol

Out of ten horses, seven completed the trial. One horse did not complete due to unavailability of the treatment remedy, while in two cases, the horse owners did not want to continue as the wounds were too painful (horse six, eight, and nine). This was unrelated to the formulation being tested. All wounds treated with the honeybee LAB formulation had a reduction in wound size and were healing effectively, or were completely healed at the end of the study (Table 1). In total, four out of seven horses had nearly complete healing, with horse one, three, and seven having complete wound closure at end of trial. In all cases, exudation was reduced and in all but two horses, pain was not obvious. In most cases, the wounds began to heal after the first application, granulation tissue was well established in some of the wounds, contraction of the wound was seen and finally epithelialization, and complete wound healing was achieved. The mean healing time was 16 days. The maturation process seemed to be very fast and even rapid hair regrowth was reported. In some cases, the wounds were not fully closed but healing was progressing (Horse five, seven, and ten). Epithelialization appeared in the wounds of horse number one at 8 days and the wounds were completely healed after 20 days (Fig. 1). The wounds on horse number two began to heal after the first treatment and were almost fully healed at 20 days. Horse number three showed immediate signs of healing and the wound closure was seen at day 16. In the case of horse number four, all wounds became smaller and were almost completely healed at the end of treatment. Horse number five had wound closure in half of the four wounds that were treated with some hair regrowth and the final two unhealed wounds had commenced healing and become smaller. The wounds of horse number seven healed in 10 days. In horse number ten, the wounds started to heal after one treatment yet had not fully healed when the study was completed (Fig. 1). No adverse effects were reported in any of the horses including in the pilot study.

### Microbial Identification

All selected isolates were identified using MALDI-TOF flex analysis or with 16S rRNA gene sequencing (Table 2). Twenty-seven bacterial genera and one yeast species (*Candida*) were identified from the wound samples with 53 identified to species level (some only to genus), the most commonly isolated belonging to *Staphylococcus* (12 species), colonizing 90 % of all wounds. Other commonly found species belonged to the following genera *Corynebacterium* (>5 species), *Streptococcus* (5 species), and *Acinetobacter* genera (>4 species) (Table 2). The majority of genera identified were gram-positive bacteria, and 56 % of all bacteria identified.

**Table 2:** Identified genus and species from all isolated bacteria and yeasts from the wound with corresponding results for in vitro antimicrobial testing

Genus	Number of infected horses (*n* = 10)	Species	Number of infected horses (*n* = 10)	In vitro inhibition with formulation
*Staphylococcus*	9	*S. aureus* *S. chromogenes* *S. delphini* *S. epidermidis* *S. vitulinus* *S. equorum* *S. schleiferi* *S. sciuri* *S. hyicus* *S. lugdunensis* *S. xylosus* *S. pseudintermedius*	7 5 4 4 2 1 1 1 1 1 1 1	Y Y Y Y Y Y Y Y Y Y^a^ Y Y
*Acinetobacter*	6	*A. iwoffii* *A. towneri* SU^b^	4 1 5	ND^c^ Y Y
*Streptococcus*	5	*S. equi* *S. dysgalactiae* *S. parauberis* *S. equinus* *S. pseudoporcínus*	4 2 1 1 1	Y Y Y Y Y
*Corynebacterium*	5	*C. diphtheriae* *C. amycolatum* *C. glutamicum* *C. casei* SU^b^	1 1 1 1 3	Y^a^ ND^c^ Y ND^c^ Y
*Arthrobacter*	*3*	*A. arilaitensis* *A. gandavensis* *A. castelli* SU^b^	2 1 1 1	ND^c^ ND^c^ ND^c^ ND^c^
*Macrococcus*	3	SU^b^	3	Y
*Aerococcus*	3	*A. viridans* SU^b^	1 2	Y^a^ ND^c^
*Psychrobacter*	3	*P. sanguinis* SU	1 2	ND^c^ ND^c^
*Bacillus*	2	*B. cereus* *B. subtilis* *B. pumilus* *B. mycoides*	1 1 1 1	Y Y Y ND^c^
*Klebsiella*	2	*K. oxytoca* complex	1	Y
*Candida*	*2*	*C. parapsilosis* SU^b^	1 1	Y^a^ ND^c^
*Aeromonas*	2	*A. bestiarum* *A. encheliea*	1 2	Y ND^c^
*Micrococcus*	2	SU^b^	2	Y
*Proteus*	2	*P. vulgaris*	2	Y
*Enterobacter*	2	*E. aerogenes* *E. ludwigii*	2 1	Y ND^c^
*Enterococcus*	2	*E. faecalis* *E. faecium*	1 1	Y Y
*Bacteroides*	1	*B. pyogenes*	1	ND^c^
*Peptonophilus*	1	*P. indolicus*	1	Y
*Pasteurella*	1	*P. canis*	1	Y^a^
*Alcaligenes*	1	*A. faecalis*	1	ND^c^
*Streptomyces*	1	*S. badius*		ND^c^
*Clostridium*	1	*C. absonum* *C. perfringens*	1 1	ND^c^
*Brachybacterium*	1	*B. faecium* *B. conglomeratum*	1 1	ND^c^ ND^c^
*Brevibacterium*	1	SU^b^	1	ND^c^
*Gordonia*	1	*G. hirsuta*	1	ND^c^
*Pantoea*	1	*P. agglomerans*	1	Y
*Carnobacter*	1	SU^b^	1	ND^c^
*Citrobacter*	1	*C. braakii*	1	Y

^a^Y corresponds to inhibition with hazy growth throughout zone

^b^SU—Species unidentified

^c^ND—Not determined

## Discussion

**Fig. 2:**
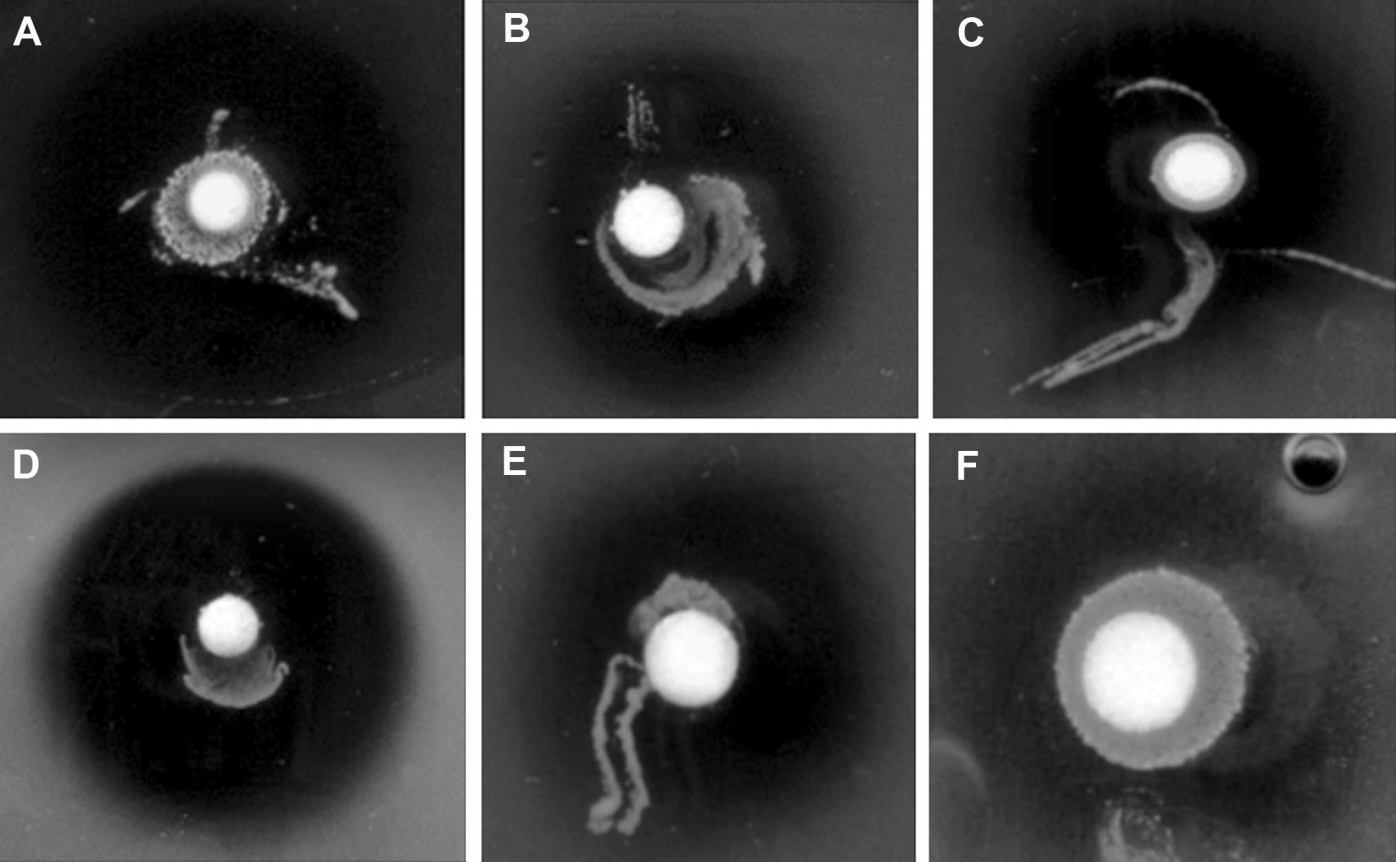
Examples of inhibition zones of the wound bacterial isolates from dual culture over lay assay, when incubated with the honeybee LAB formulation: *Bacillus subtilis* (**a**), *Streptococcus equinus* (**b**), *Staphylococcus aureus* (**c**), *Bacillus pumilis* (**d**), *Staphylococcus equorum* (**e**), and *Enterococcus faecium* (**f**)

Like human chronic wounds, equine wounds can be extremely difficult to heal due to many different factors [59]. Some have suggested that the healing capability of equine wounds are very similar to human wounds and that they are an important wound model to investigate in regards to wound management [51]. Many treatment options have been investigated in the past few years for treating hard-to-heal wounds; however, none of them seem to be optimal for an effective management [32, 58]. Honey as a wound dressing provides a moist environment but also antibacterial and anti-oxidative action from the presence of high osmosis, hydrogen peroxide, and other substances [15, 26, 30]. It has also been reported to reduce swelling and inflammation and can decrease the healing time by stimulating angiogenesis, granulation, and epithelialization [2, 15]. Studies have claimed Manuka honey to be effective in equine wound healing [21] including a study showing Manuka honeys effect on reduction in wound size yet at 35 days none of the wounds were fully closed [7]. In the case of our study, we saw some of the wounds close in a short space of time (Table 1), suggesting the LAB and honey together have added benefits for wound healing than just honey on its own. Heather honey was also shown to be effective against bacteria associated with equine wounds in comparison to other honey types [13]. This is possibly due to the slightly higher water content in heather honey than in other honey types, which allows for greater activity of the LAB substances and their viability. Another study has also shown the effectiveness of a hydrogen peroxide (H_2_O_2_) topical ointment in the treatment of equine wounds [52] which could explains honeys action. H_2_O_2_ is produced in large quantities by the LAB symbionts [33] and by the honeybee itself that is inactivated during honey ripening, but then reactivated when diluted by the wound exudate leading to its slow release [3]. This is why we believe that honey is the optimal treatment medium for its beneficial properties in wound healing and for the survival of the LAB symbionts that need food (honey) to multiply and produce bioactive substances [45, 56]. Previous research has demonstrated that many of the specific therapeutic properties of honey are attributed to the 13 LAB symbionts used in this study, for instance *L. apinorum* Fhon13 produces 2-heptanone which has anesthetic qualities [42], five LAB strains (*L. apis* Hmall, *L. kimbladii* Hma2, *L. melliventris* Hma8, *L. helsingborgensis* Bma5, and *L. kullabergensis* Biut2) produce H_2_O_2_, and the production of hydroxy fatty acids by *L. apinorum* Fhon13, *L. kunkeei* Fhon2, *Bifidobacteriumasteroides* Bin2, and *Bifidobacterium* species Bin7. [41]. Remarkably, these 13 LAB symbionts are viable and active in large quantities in fresh honey (approximately 10^8^ per gram fresh honey, depending on the honey type) [39, 54, 56] and are one of the main contributors’ to antimicrobial activity associated with honey due to their production of several bioactive substances that build up this defense against pathogens making it impossible for them to survive [41]. This can be observed from the in vitro experiments both in this study and in previous studies [30, 37], in which the antagonistic action against environmental and wound pathogens of honeybee LAB [33] in combination with honey [34] is very effective (Fig. 2). On the other hand, the matrix composed of only the heather honey with no viable LAB had no antimicrobial action [12].

In the case of equine wounds, a short healing time is extremely important as horses will be exposed to the outside hostile environment like moist mud and grass, in which exposure to microbes is certain. The diagnosed Pastern Dermatitis wound that horse number one had was healed completely in 20 days. Pastern Dermatitis is diagnosed as a syndrome more so than disease due to the wide variety of genetic and environmental factors that exacerbate the condition making it difficult to manage [18]. The small wounds of horse number two, healed significantly slower compared to the other horses (Post study follow up with owner revealed wounds had healed at day 30). However, the reasoning for this could be that this horse was much older (23-year old) and had an underlying condition of chronic lymphangitis, which caused substantial inflammation and edema in the limbs. Horse number ten showed a slow healing time that could be explained by the long duration of this wound (4.5 years). Even though many of the wounds were slow to heal, we saw that in some cases the wounds healed in a short amount of time compared to studies that used honey alone where only reduction in wound size is seen [6, 7].

In the case of horse number seven we noticed the wounds healed in 10 days, which according to the owner was the first time they were healed since in 20 months. Interestingly this wound showed the presence of *Candida*, a yeast, as well as *Staphylococcus* species (Table 2), suggesting the honeybee LAB formulation may have variation in its antimicrobial action against both yeasts and bacteria. Previous results have shown that the LAB species produce different inhibitory patterns when in contact with different bacteria and yeasts and the level of activity is species dependent, with some more effective than others [23, 41, 56]. The different LAB species produce a great variety of substances in which some could be more effective against yeasts than bacteria, for example, *L. apinorum* Fhon13 produces 3-hydroxy fatty acids that have been shown to have antifungal activity [47]. Additionally the LAB strains produce large amounts of organic acids which have multiple functions including acetic acid which is known to be effective against *P. aeruginosa*, a significant wound pathogen [38]. All these bioactive substances in combination together make a formulation with the viable and co-working LAB species that can produce these substances in the wound environment when needed, making them a very attractive tool against a wide range of pathogens [30, 36, 37]. This broad spectrum antimicrobial activity of the LAB is perfect for the use in wound management. As like human wounds, we observed the equine wounds are colonized by multiple species and genera at the same time, and these LAB symbionts can work synergistically in the wound environment. Similar to human chronic wounds, we observed that all wounds were colonized by more than one bacterial species (Table 2).

We observed 90 % of horses were colonized by *Staphylococcus* species (Table 2). *Staphylococcus* species are a very significant wound pathogen in both humans and animals, and it is believed by many that *S. aureus* and other species are heavily involved in the hard-to-heal nature of equine wound infection [13, 57]. Due to the global increase of antibiotic-resistant strains such as MRSA and *β*-lactamase producing pathogens, development of alternative treatments for both human and equine wounds is urgent [10, 14]. Furthermore, animals infected with MRSA and other antibiotic-resistant pathogens have also increased in the last decade [5, 57] and are now often reported by veterinary practices and farm owners [4, 57]. Previous research points toward the utilization of these newly characterized LAB symbionts as a new antibiotic alternative due to their antimicrobial effect against some antibiotic-resistant pathogens found in humans, including MRSA [41, 56].

It is clear from the wide microbial variety identified from the equine wounds that many could be causing the chronic symptoms associated with non-healing [58, 59]. Several possibly colonizing the wound from their natural habitat (soil, grass) e.g., *Acinetobacter* species [24]. A review of research by Westgate and colleagues summarize that generally gram-positive bacteria make up the common equine skin flora which is significant to the horses health but in chronic wound colonization, these bacteria can act as opportunistic pathogens e.g., *S. aureus* and *Streptococcus* species [58]. It does seem when looking at this study, however, that *Staphylococcus* species are at the forefront of chronic wound infection in horses.

This was a small pilot study aimed at investigating potential microbial reduction in wound bacteria and stimulation of wound healing in an animal model. It was also aimed at detecting potential adverse effects in which we can now confirm that the formulation is not harmful in the case of topical application on horses. It is important to not also that these were “real” wounds and not manufactured as seen in some studies [7], therefore represent a more realistic infection for the horses. A placebo-controlled study comparing this formulation is the next step to confirm these results and investigate the possibilities to apply the formulation in humans. In this study, each wound was its own historical control. Previous unsuccessful treatments (Table 1) of these wound also serves as a reference and strongly demonstrate the significance of the new type of treatment used in this work.

## Conclusion

The rapid, painless healing of hard-to-heal equine wounds gives us reason to believe that the honeybee LAB formulation presents a new topical option in future wound healing. This new treatment may be a stepping-stone toward an alternative solution for treating other infected wounds in animals and humans and warrants further investigation.
